# Segmentation of Brain Tumor Using a 3D Generative Adversarial Network

**DOI:** 10.3390/diagnostics13213344

**Published:** 2023-10-30

**Authors:** Behnam Kiani Kalejahi, Saeed Meshgini, Sebelan Danishvar

**Affiliations:** 1 Department of Biomedical Engineering, Faculty of Electrical and Computer Engineering, University of Tabriz, Tabriz 385Q+246, Iran; b-kiani@tabrizu.ac.ir; 2 Department of Electronic and Computer Engineering, Brunel University, London UB8 3PH, UK

**Keywords:** generative adversarial networks, brain tumor, medical image segmentation, computer aided diagnosis

## Abstract

Images of brain tumors may only show up in a small subset of scans, so important details may be missed. Further, because labeling is typically a labor-intensive and time-consuming task, there are typically only a small number of medical imaging datasets available for analysis. The focus of this research is on the MRI images of the human brain, and an attempt has been made to propose a method for the accurate segmentation of these images to identify the correct location of tumors. In this study, GAN is utilized as a classification network to detect and segment of 3D MRI images. The 3D GAN network model provides dense connectivity, followed by rapid network convergence and improved information extraction. Mutual training in a generative adversarial network can bring the segmentation results closer to the labeled data to improve image segmentation. The BraTS 2021 dataset of 3D images was used to compare two experimental models.

## 1. Introduction

Various imaging methods in medicine are designed for specific purposes, among which the most widely used are angiography (using X-rays), computer tomography (CT) scans (using X-rays), sonography (using ultrasound waves), magnetic resonance imaging (MRI) (using radio waves and magnetic amplification), and radiology (using X-rays). Each of the mentioned imaging techniques is specific to certain tissues in the body. The MRI technique provides clinicians with crucial information on the type, size, shape, and location of brain tumors without exposing the patient to dangerous radiation. Meningioma and glioma are fatal brain tumors that can be discovered via magnetic resonance imaging studies [1]. If tumors are not diagnosed in their early stages, they can be seriously dangerous in the future. T1, T1c, T2, and FLAIR MRI sequences provide detailed information about brain tumors:

(1) T1-weighted scans that distinguish healthy tissues from those with tumors.
(2) T2-weighted scans to delineate the tumor area, which creates a bright image area.
(3) T1-c scans use a contrast agent that builds up at the edge of the tumor and gives a bright signal.
(4) The water molecule suppression signal is used in FLAIR scans.

Glioma is the most common kind of brain cancer in humans. The World Health Organization (WHO) divides tumors into four types [1]. Low-level tumors such as meningioma are classified as grade one and two cancers, whereas gliomas are classified as grade three and four cancers. Meningiomas account for around 20% of all brain tumors. This type of tumor has a spherical shape and grows at a slower rate. Even though meningioma is a low-risk tumor with a modest growth rate, it can cause considerable harm to the patient if not treated early. Since lesions are typically tiny and have varying color, shape, and texture alterations, interpreting MRI images to detect brain tumors is a time-consuming, difficult, and delicate process. Neurologists and surgeons sometimes struggle to make the right call. Noisy images and exhausted doctors can also cause misinterpretations of images. Analysis with the help of computer algorithms is one of the most promising methods for facing such problems in MRI images. In the meantime, deep learning (DL) architectures are prominent as a method and work well in this field.

### 1.1. Brain Tumor Segmentation

The present categorization of methods for brain tumor segmentation can be classified into many groups according to unique conceptual frameworks. The categorization of brain tumor segmentation methods is commonly based on the level of human involvement, resulting in three distinct categories: manual, semi-automated, and fully automatic [2]. Proficient manual brain tumor segmentation requires professionals to possess a comprehensive understanding of picture interpretation, brain tumor characteristics, and relevant disciplines such as anatomy. The manual segmentation of brain tumors is the process of manually delineating the boundaries of the tumor and assigning distinct labels to the various regions of the anatomical components. To date, manual segmentation has been extensively utilized in clinical trials. Due to the association of numerous images with the progression of brain tumors, professionals may encounter challenges when manually segmenting various regions of brain tumors, as this process is prone to errors, time-consuming, and yields unsatisfactory results. Consequently, the utilization of more sophisticated segmentation techniques, such as semi-automatic and fully automatic segmentation methods, can effectively address this issue. The process of semi-automated brain tumor segmentation requires the utilization of specialized knowledge, human involvement, and computational tools. In the context of brain tumor diagnosis, semi-automated procedures involve the input of parameters by an expert, the analysis of visual data, and the provision of feedback for software computations. The method category is comprised of three distinct components: initial processing, feedback, and assessment. While semi-automated algorithms for brain tumor segmentation have demonstrated superior performance compared to manual segmentation, it is important to note that discrepancies in findings can arise due to variations among various specialists and over different time points. Consequently, there has been a development of techniques aimed at achieving the fully automated segmentation of brain tumors.

### 1.2. Deep Learning

Deep learning is a subfield within the broader discipline of Machine Learning (ML) that is employed to model complex problems and abstract notions. Deep learning (DL) facilitates the training of models that consist of numerous layers of processing, known as deep neural networks, which have the ability to acquire abstract representations of data. The assessment of conceptual qualities as nonlinear functions of low-level features is facilitated by the multilayer nature of DL networks. Convolutional Neural Networks (CNN), Restricted Boltzmann Machines (RBM), Deep Belief Networks (DBN), Deep Auto-Encoders (DAE), Recurrent Neural Networks (RNN), and their derivatives, including Long Short-term Memory (LSTM), are considered to be very valuable Deep Neural Networks (DNNs). These networks are frequently employed to execute extensive operations for a multitude of objectives. The important difference between conventional machine learning (ML) and deep learning (DL) algorithms is due to the aspect of feature engineering. Traditional machine learning (ML) algorithms perform classification tasks by utilizing a predefined set of characteristics. In contrast, deep learning (DL) techniques possess the ability to automatically extract features, resulting in higher accuracy compared to conventional ML models. The effectiveness of these models in addressing large-scale data issues surpasses that of shallow machine learning techniques due to the increased depth of the processing layers [2]. The study applied various machine learning (ML) methodologies, such as the random forest algorithm and Support Vector Machines (SVM), to automate the process of identifying and segmenting lesions using MRI data. Deep learning methods such as Restricted Boltzmann Machines (RBM), Denoising Autoencoders (DAE), Convolutional Neural Networks (CNN), and Long Short-Term Memory (LSTM) have recently been increasingly prominent in the field of medical picture analysis. The Generative Adversarial Network (GAN) is a widely recognized and very efficient deep learning model.

### 1.3. GAN Network 

**Figure 1:**
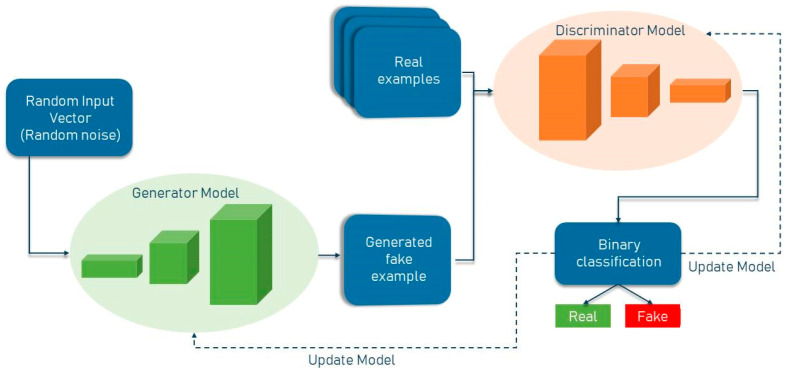
General structure of GAN network.

GAN networks are generative models that generate high-quality data from a small collection of training datasets. The GAN network is made up of two components: the generator and the discriminator. The generator attempts to learn the model built from the data and, as a result, generates graphics from random noise inputs. The discriminator is a CNN network that attempts to discriminate between actual data (training data) and data generated by the generator, and it assesses the likelihood of a mistake using this approach. Two of the most well-known GAN architectures are the semi-supervised GAN and Laplacian pyramid GAN. Since GAN networks are trained using only non-lesion images, it is expected that the error probability assigned by the network to images with lesions will be significantly different from that assigned to non-lesion images. The general structure of a standard GAN network with a generator and discriminator is shown in Figure 1. In general, GANs have two uses in medical imaging. The first use is concerned with generation, which can aid in understanding the underlying structure of training data and learning to produce new pictures. This aspect of GAN networks holds great promise for coping with data shortages while protecting patient privacy. The second application emphasizes the differentiating feature. Due to the necessity for vast volumes of training data, image creation methods have proliferated in the field of deep learning. Recent research has demonstrated that GAN networks may be utilized for a variety of applications, including image-to-image translation [3] and unsupervised representation learning. GANs have also been demonstrated for unsupervised data domain pattern matching in multimodal medical imaging, indicating their potential for usage with limited medical imaging data.

This research is organized into the following sections: Section 2 of this paper presents an overview of the relevant research and studies conducted on the architecture of Generative Adversarial Networks (GANs), covering various methodologies and technical foundations. The configuration of the suggested framework is detailed in Section 3, while the evaluation of the test results will be discussed in Section 4. Section 5 is the Concluding Reflections on the Proposed Research.

## 2. Review of Previous Works

The generation of labeled data to train a deep network for brain tumor segmentation in MRI scans is time-consuming and requires the contribution of experienced radiologists.

### 2.1. CNN and FCN Networks Methods 

Dong et al. demonstrated an FCN network for detecting and segmenting brain tumors. Some instances of low-grade glioma were less successful. The U-Net hybrid pyramid model was proposed by Kong et al. [4]. The U-Net method examines both general and regional data. Alex et al. [5] proposed utilizing an FCN network to segregate brain tumors. To eliminate false-positives, they used a 23-layer voxel-based classification using a forward path and linked component analysis. Hawai et al. [6] improved brain tumor segmentation on BraTS. Their network was two-way and cascaded. Two-way architecture considers two receiving portions’ local and global properties. Wang et al. [7] employed convolutional neural networks in a cascade architecture to automatically segment brain tumors. Hossein et al. [8] employed deep cascade neural networks to segregate brain tumors. Hyunwoo et al. [9] presented a decoder–encoder network for semantic segmentation that aggregates high-resolution data into low-level salient edges. 

In 2015, Ronberger et al. [10], inspired by the DL approach, proposed a U-Net architecture upon fully convolution networks and used mirror concepts to predict border pixels. The basic idea behind this network is to augment a decreasing route with regular convolutional layers, where merge operators are substituted by upsampling operators. As a result, these layers improve the output resolution. For precise localization, the contraction path’s high-resolution characteristics are mixed with the upsampled output. Then, a successive convolution layer can learn to provide a more accurate output based on the information. In this architecture, the left side is the contraction side, the center is the bridge or bottleneck, and the right side is the expansion side. The three large gray arrows show how the learned feature mapping is copied from the contraction path to the expansion path. This transfer of learned features is the key point of U-Net, which gives the segmented image.

Inspired by U-Net, Miltri et al. [11] presented V-Net for brain tumor segmentation using MRI data. This network has a shrinking path with convolution layers and 5 × 5 × 5 volume kernels for downsampling and a dilation path for deconvolution to increase data size. This helps segmentation. V-Net is an extension of U-Net’s 3D architecture, and each convolution and deconvolution layer are volumetric.

Low- and high-grade gliomas have different architectures. Post-processing volume threshold limitations eliminated the inaccurate cluster categorization error. During training and assessment, the following methods clustered medical scans. SegNet was created by Drozdahl et al. Instead of long connections, they used identity mappings (short-hop connections) [12]. These identity maps allow for a deeper vanishing gradient CNN network and quick learning by recovering lost spatial information during dimensionality reduction. The researchers designed a deeper network with a small core. Pereira et al. [13] constructed a 33-kernel DNN. This model’s fewer weights minimized overfitting. Kamenisas et al. [14] reported a fully linked 3D neural network for brain lesion segmentation. Cui et al. [15] employed a deep cascade neural network to segregate brain tumors. Lin et al. [16] optimized segmentation using fully connected conditional dense learning and CNN. Zhao et al. [17] integrated FCN networks to improve tumor segmentation. Badrinarayanan et al. [18] proposed SegNet, an encoder–decoder architecture. In this architecture, maximal aggregation indices contain all the information in floating precision without feature maps, and the accompanying encoder upscales the input feature maps. Raza, R. et al. [19]’s proposed model is a hybrid of the deep residual network and U-Net model (dResU-Net). The residual network is used as an encoder in the proposed architecture with the decoder of the U-Net model used to handle the issue of the vanishing gradient. The proposed model is designed to take advantage of low-level and high-level features simultaneously in order to make the prediction. Yousef, R et al. [20] introduced Bridged U-Net-ASPP-EVO which exploits Atrous Spatial Pyramid Pooling to enhance capturing multi-scale information to help in segmenting different tumor sizes, Evolving Normalization layers, squeeze and excitation residual blocks, and the max-average pooling for downsampling in two variants.

### 2.2. GAN Networks Methods 

As a result of the preceding, a model that takes all the images as the input and utilizes local and global features to balance the data’s class imbalance is required. To produce high-quality results when analyzing medical images, methods based on GAN networks are frequently used. The following studies, each of which has advantages and disadvantages that will be discussed below, are related to and are like the subject of this study. Different architectures characterized low- and high-grade gliomas, post-processing volume thresholds eliminated cluster categorization errors, and cluster medical scans were carried out during training and assessment. Zhu et al. [21] demonstrated unpaired image training for style transfer. This approach removes fog from photos, adds and removes particles, and more. Ref. [22] proposes a two-stage GAN network (ToStaGAN) to improve brain tumor segmentation. ToStaGAN is a two-stage GAN that uses high-level semantic knowledge to improve brain tumor segmentation. In ToStaGAN, the U-Net network is adopted as the “coarse” generating network in the first step, and a U-shaped text auto-encoder (ConEnDer) is offered as an “excellent” production network in the second stage. This approach improves semantic segmentation by raising numerous parameters. ConEnDer can collect more diversified and abstract characteristics, and by using FEM and coarse prediction map features from the previous stage, it may produce optimal segmentation results. Two-stage production is superior to one-stage. ToStaGAN outperforms the rival segmentation methods. Ref. [23] offers a brain tumor segmentation technique based on GAN. A 3D U-Net is used for segmentation and a classification network is used for discrimination in the GAN architecture. Both integrate multidimensional context information using 3D convolutions. The U-Net 3D model’s extensive connectivity improves network convergence and accuracy. Adversarial training improves segmentation results by bringing them closer to the labeled data, allowing the network to segment unexpectedly tiny tumor subregions and reliably identify each voxel. The suggested method precisely segments brain tumors. In addition, [24] creates the RescueNet network architecture by combining residual and mirror principles. CycleGAN [21], which is used for image-to-image pattern transfer, is improved by this design. For improved brain MRI segmentation, this technique employs less training data and a non-pairwise strategy. RescueNet uses unpaired adversarial training to better the overall tumor, then the central portion of the brain MRI. Preparing vast amounts of labeled data for deep network training is time-consuming and labor-intensive. To train the proposed network without paired data, unpaired training is utilized. In [25], the unsupervised computer modeling of symmetrical alterations in normal brains is used to separate brain cancers from MRI pictures without symmetry. SD-GAN is an unsupervised brain tumor segmentation algorithm. The SD-GAN model learned non-linear mappings between left and right brain pictures to describe normal brain variability (symmetry). The trained SD-GAN was then used to recreate normal brains and segment brain tumors based on asymmetry. Two benchmark datasets were used to test SD-GAN. SD-GAN outperformed new unsupervised segmentation algorithms and was comparable to supervised U-Net. This study showed that symmetric alterations (intrinsic anatomical changes) can be predicted using unannotated normal MRI data and employed in tumor segmentation. Ref. [26] compares rapid MRI algorithms that leverage GAN using anatomical data. This is done to prove that rapid MRI is generalizable and trustworthy, and to look ahead.

In [27], a GAN-based MS-GAN semantic segmentation framework is suggested to localize MS lesions in multimodal brain MRI. It comprises a multimodal encoder–decoder converter G and numerous classifiers D. Multiple input methods are employed to create the generator, which bypasses the encoder’s location information to the decoder to reduce network parameters and improve localization performance. The converter combines multimodal imaging data with multipath encoding and cross-combination. An additional category-related constraint is presented for GAN model adversarial training to reduce convergence in category-based image-to-image translation challenges. In total, 126 relapse MS individuals were evaluated. Performance was compared to other semantic segmentation models including patch-based deep learning. This method segmented more accurately than the advanced techniques. In [28], a new automatic data augmentation approach using GAN networks for reinforcement learning is described, allowing a machine learning-based method to learn current annotation instances more efficiently. This architecture uses a coarse-to-fine generator to generate generic enhanced data from training sets. In trials, the methodology improves MRI pictures by 3.5% compared to typical enhancement methods using the BRATS15 dataset. Using a collaborative segmentation network improves the BRATS15 performance. Ref. [29] proposes an end-to-end GAN-based technique for brain tumor segmentation. This method combines generative and discriminative models and uses GAN instead of conditional random fields for high-order smoothing (CRF). The proposed technique was evaluated in the BRATS 2015 database, and it was found that using GAN enhances the network performance. This method separates a patient’s brain tumor image in 10.8 s less than the existing CNN-based methods. In [30], it is evaluated if synthesizing brain MRI data with GANs allows the employment of a DL model for segmenting T1-weighted, post-contrast T1-weighted, FLAIR, and T2-weighted brain lesions. In this study, 2011–2019 brain MRI data were gathered and simulated without T1-weighted and FLAIR images. Two GANs were trained, verified, and tested on 210 glioblastomas (BRATS 2017) to create T1-weighted pictures from postcontrast T1 images and FLAIR images from T2-weighted images. MSE and SSI were used to evaluate image quality (SSI). A dice similarity coefficient was used to compare segmentations from produced and original MRI scans (DSC). GANs were verified on GBMs and CNS lymphomas to test generalizability. Mann–Whitney, Friedman, and Dunn tests were used. When MRI sequences are absent, GAN-generated brain MRI scans can be fed into deep learning models. In [31], a deep learning approach for classifying MRI tumors is described. A deep neural network is trained as a discriminator in a GAN’s convolutional layers on distinct MRI datasets to extract robust features and understand the visual structure. After replacing completely linked layers, the deep network is taught to recognize three types of tumors. There are six layers in all, with 1.7 million weight parameters. Pre-training a GAN as a discriminant, along with data augmentation (image rotation and mirroring), reduces over-training on a small dataset. This approach was used on 3064 T1-CE MRI scans from 233 people. A neural network in a GAN is initially trained as a discriminator in its convolutional layers on multiple MRI datasets to extract robust features and grasp the structure of MRI pictures. After replacing completely linked layers, the deep network is taught to recognize three types of tumors. There are six layers in all, with 1.7 million weight parameters. Pre-training as a GAN discriminant combined with data augmentation and deletion reduces over-training on a small dataset.

In [32], Kiani Kalejahi, B. et al. used the possibility of producing multiple-sequence MR images by the application of auxiliary classifier-generating adversarial networks (ACGANs). At the beginning, a deep neural network is trained to function as a discriminator in GAN data sets consisting of MRI images in order to extract the features and also learn the structure of the MR images in their annular layers, and then the layers that are already fully connected are removed.

## 3. Suggested Method

A neural network is proposed and developed based on the electrochemical activities of neurons. The task of these networks is to model human neural activities in learning knowledge and apply the knowledge obtained in similar situations. Various models have been proposed for modeling neural structures, among which structures like CNN have performed better in modeling the activity of the human visual system in relation to the visual cortex of the brain. The proposed method of this research attempted to design and improve the optimal structure of this combination of these two types of networks for the segmentation of brain MRI images by utilizing innovations related to CNN and GAN networks in various topics of machine vision over the last few years.

This approach develops a GAN framework where one neural network (the generator) tries to generate a model that resembles real neuron activity. In contrast, the other network (the discriminator) tries to distinguish between real and generated data. In addition, it defines a loss function that incorporates both the traditional GAN loss (adversarial loss) and a loss related to the generated model to the real data and fine-tunes the network architecture and hyperparameters based on the performance of a validation set.

### 3.1. Data Preprocessing

The term “data preprocessing” is used to describe the process of preparing data for use in training a network. Image resizing, or scaling volumetric images to a specified dimension, is a useful preprocessing technique in this situation. As datasets might contain information on a wide range of dimensions, it is vital to standardize the data across all of them. So, resampling the images to get a consistent image resolution is important. This easy technique can also assist in keeping the number of calculations from increasing excessively when large images are delivered over a network. The term “normalization” refers to a crucial step in the pre-training phase of neural network image processing. Normalization is used to adapt measurements to a standard scale. When the inputs to a neural network have significantly different scales (the pixel values of the pictures are of varying sizes), normalization is necessary. Standard normalization procedures include min–max [12], which shifts the input range to a new interval (often 0–1), and z-score [13], which transforms each input sample so that its mean is zero and its variance is one. The use of a decimal system of measurement is highlighted [14]. The min–max technique is used to standardize the raw data in this research.

### 3.2. Structure of the Proposed Method

A fully convolutional network (FCN) is combined with a generative adversarial network (GAN) in the suggested method. The proposed GAN network design has two major nodes: a generator and a discriminator.

- Generator: A segmentation network that automatically creates segmented images of brain tumors. This is referred to as the algorithm’s core module.
- Discriminator: the classification network validates the input, which indirectly improves segmentation accuracy.
- Loss function: adversarial training coordination between generator and discriminator, combination of conventional segmentation and adversarial training optimization objectives.

Once the network is randomly set up, its parameters stay the same. The generative network used for segmentation is then repeated to create a generative network that does not need adversarial training. The parameters of the generator network are then modified, and the discriminator network is repeated. The alternative loop iteration method can generate segmentation results while enhancing classification accuracy.

As previously stated, the suggested 3D CNN is based on the FCN approach and is employed in two structures: the generator and the discriminator. FCN networks such as [15], 3D U-Net [16], and V-Net [11] have seen substantial growth in medical image segmentation applications in recent years. The FC layer(s) in a traditional CNN are totally deleted in the FCN method, and convolution layer(s) are employed instead. In general, the selection of the FCN network for segmentation applications is a very difficult and complex task. There are some networks in the literature (such as [17] and [18]) that are used for this application. These networks, however, do not provide the necessary interdependence at the ground level. Adding hop connections between the upsampling and downsampling pathways is how the U-Net architecture [10] gets around this issue. The word “mirroring” has come to describe this type of alteration, and gradient fading is another issue with the training of deep networks. Residual Network Architecture [7] proposes using homogeneous mappings, or shortcut paths, to solve the issue of gradient fading during back propagation and learning network parameters.

**Figure 2:**
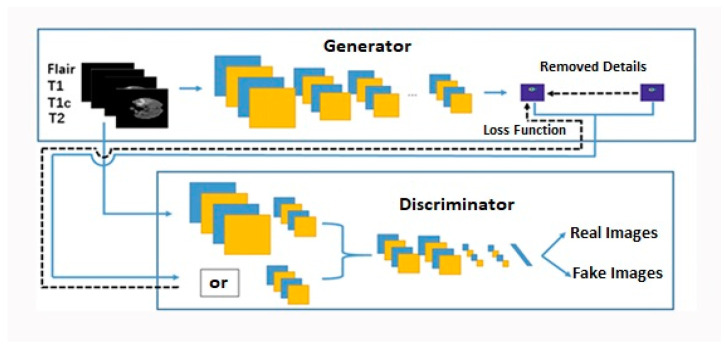
The general structure of the proposed GAN network. Direct diffusion is shown using blue lines and reverse diffusion is shown using black dotted lines.

A distinct Fully Convolutional Network (FCN) architecture has been developed for the purpose of segmenting brain tumors. This architecture is specifically designed for the two components of the proposed Generative Adversarial Network (GAN), namely the generator and discriminator. The suggested Generative Adversarial Network (GAN) incorporates the ideas of mirroring and residual. Figure 2 illustrates the comprehensive architecture of the proposed Generative Adversarial Network (GAN). The model consists of two distinct sub-networks, namely the generator and the differentiator, each with its own individual framework. In order to address the issue of image generation, the traditional GAN network architecture is employed, wherein the generator component is utilized to generate synthetic images through the algorithmic process. In order to address the image segmentation problem, the GAN network generator model is employed by replacing the conventional generator with the image segmentation network. The generative network is capable of autonomously segmenting images that are classified as unreal. Furthermore, the discriminator can serve the purpose of segregation and classification. Numerous sophisticated models are built upon Convolutional Neural Networks (CNNs) that are specifically designed for the purpose of picture classification. Nevertheless, there are fundamental differences between dense prediction applications, such as semantic segmentation, and picture classification. In this study, we employ a novel convolutional module known as extended convolution [33], specifically designed for the purpose of dense prediction. The module offered utilizes an extended convolution technique to gather multiscale conceptual information while minimizing any loss in resolution. Extended convolutions enable a significant expansion of the affected region, exponentially increasing its scope, while maintaining high resolution and comprehensive coverage. The study conducted by Yu et al. [33] provided evidence of the module’s ability to improve the performance of semantic segmentation systems. The utilization of extended convolution has been implemented for all convolutional layers in this study based on the provided justification and empirical observations.

#### 3.2.1. Generator Structure

The design provided for the generating structure in our research involves the utilization of a three-dimensional fully convolutional network (3D FCN) to perform the multi-class segmentation of magnetic resonance imaging (MRI) images showing brain tumors. The FCN architecture we propose integrates the U-Net model with the Residual Network. The suggested Fully Convolutional Network (FCN) is designed to accept three-dimensional (3D) Magnetic Resonance Imaging (MRI) pictures of varying dimensions as its input. The segmentation map generated offers the probability distribution of the voxel classes at each spatial position. The quantity of channels depicted on the map is equivalent to the quantity of labels present. The network depicted in Figure 2 consists of two distinct pathways: the Downsampling path, sometimes referred to as the straw sampling road, and the Upsampling path, also known as the rising sampling path. The convolution operation consists of a series of sequential layers, including volume convolution, normalization, and activation. In addition, the construction of feature jump links is implemented to enhance the segmentation feature maps’ quality by facilitating the transition from the contraction path to the expansion path.

**Figure 3:**
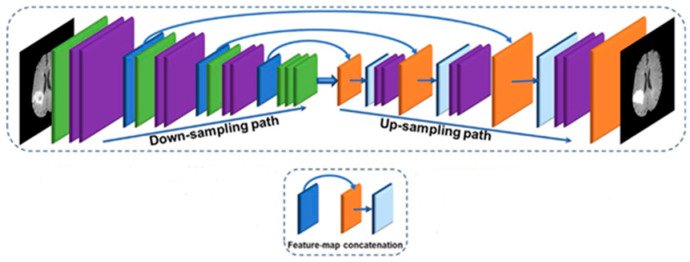
Suggested 3D-GAN network generator portion architecture’s general structure.

The generator network under consideration comprises three distinct building blocks, namely a convolutional block, a residual block, and an output block. The composition of each CB block consists of a three-dimensional extended convolution layer, a group normalization (GN) layer, and an exponential linear unit (ELU) activation function. In contrast, every database block consists of a three-dimensional extended deconvolution (Deconv) layer, a group normalization (GN) layer, and an exponential linear unit (ELU) activation function. In order to achieve feature compression sampling, the integration of residual blocks is implemented at intermediate phases of the contraction process. The aforementioned blocks are designed to tackle the issue of gradient fading that arises in the process of back-propagation. Within the framework of the expansion pipeline, it is observed that an intermediate stage exists whereby an upsampling layer is implemented prior to the subsequent appending layer. The layer serves as a connection between the expansion path feature maps and the contraction path feature maps, as their quantities may vary. Additionally, the incorporation of this topology adds a sense of symmetry to the network under consideration. To address the problem of resolution loss, a concatenation layer is included between the symmetric feature maps obtained from the contraction path and the output of each Deconv layer in the expansion path. The information pertaining to the quantity and sizes of feature maps, which provides a more detailed understanding of the network’s intended functionality, is shown in Figure 3.

The generative network generates a segmentation volume that matches the dimensions of the input volume. The number of final convolutional features in the generated volume is determined by the desired number of classes for prediction, achieved through the use of 1 × 1 × 1 convolutions. It is worth mentioning that the study of volumetric images necessitates the utilization of 3D operations, such as convolution, deconvolution, and fusion layers. These operations are presently supported by some machine learning frameworks, such as TensorFlow. The aim of the subsequent sections is to provide clear definitions of these notions in order to enhance the comprehension of the proposed network.

#### 3.2.2. Discriminating Structure

The utilization of the 3D FCN network in the GAN network involves the categorization of images as a discriminator, which in turn generates the generative model. The primary purpose of this network is to differentiate real input from fake input. The discriminator segment inputs a pair of images consisting of the initial segmentation images and the images segmented by the generator segment. Based on the generated segmented images, it is apparent that this pair of images may be classified into two separate groups:

(1) Experts manually label original images and segmented images. In other words, these categories are segmentation masks and represent the real value and the category with the value.
(2) The generator automatically labels both the original image and the segmented image with the generated value. The category is 0.

#### 3.2.3. Convolutional Neural Networks

The convolutional layer is used as the primary component within the Convolutional Neural Network (CNN) architecture, responsible for conducting convolutions between numerous filters, commonly referred to as “kernels,” and the input image. The filters execute the convolution process by traversing the input with respect to its dimensions. The result derived from this process is sometimes referred to as a “feature map” or an “activation map.” In a more precise style, the convolutional layers of a neural network receive the unprocessed image as the input in order to compute the resulting signal. This enables the deep learning model to circumvent the need for manual techniques in the segmentation of brain tumors within MRI brain images. The resolution of intricate abstract learning patterns can be effectively achieved by the utilization of non-linear algorithms and varying degrees. The objective of utilizing this layer is to extract features from input images. This is achieved by convolving the pixels of the input image with a small region known as the receptive field, which exhibits local connectivity. Indeed, it might be posited that this stratum imparts a sense of profundity to the visual representation. The hyperparameters of the system consist of a collection of trainable filters, which encompass the dimensions (F) and step size (S) of each filter. The term “step size” (S) denotes the quantity of pixels by which the window is displaced following each action. In the majority of instances, SF is regarded as being significant. It is noteworthy to emphasize that the convolution process can be executed in one-dimensional, two-dimensional, and three-dimensional formats. In the network under consideration, the utilization of a 3D convolution operation is necessitated by the inherent three-dimensional structure of the input images. As previously stated, a specialized convolutional module known as extended convolution has been employed for conducting 3D convolution operations. One notable advantage of this method in comparison to the traditional convolution operation is its ability to provide a broader perspective with a reduced number of parameters. This enables the extraction of a greater amount of content information from the input, resulting in enhanced efficiency. Next, the difference between extended convolution and standard convolution will be explained.

(1) Let $F\;:\;Z^{2}\to R$ be a discrete function;
(2) Let $\Omega_{r}={\left[-r,r\right]}^{2}\cap Z^{2}$;
(3) $k\;:\;\Omega_{r}\to R$ is a discrete filter with size ${\left(2r+1\right)}^{2}$.

If it is assumed that these conditions are true, then the discrete convolution encoder can be defined as a relation:

(1) $$\left(F*k\right)(p)=\sum_{s+t=p}F\left(s\right)k(t)$$

Now, the generalized state of this operator can be defined as relation:

(2) $$\left(F{*}_{l}k\right)(p)=\sum_{s+lt=p}F\left(s\right)k(t)$$

where *l* is the expansion coefficient and ${*}_{l}$ is the expanded convolution operation. It is worth noting that the standard convolution operation is a special case of extended convolution operation when *l* is equal to one.

This operator is based on the notion that extended convolution expands the region of impact exponentially without sacrificing coverage or image resolution.

If $F_{0},F_{1},\ldots ,F_{n-1}\;:\;Z^{2}\to R$ are discrete functions and $k_{0},k_{1},\ldots ,\;k_{n-2}:\;\Omega_{1}\to R$ is a discrete filter with a size of 3 × 3, then the filters are applied by expanding exponentially for *i* from *0* to *n* − 2 in the form of the relation:

(3) $$F_{i+1}=F_{i*2^{t}}k_{i}$$

**Figure 4:**
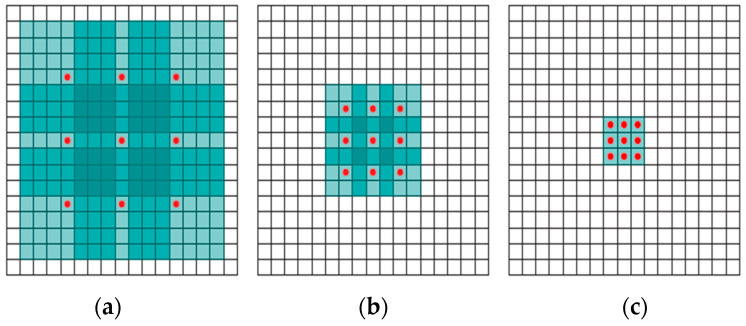
Systematic expansion allows for the exponential extension of the impact area while maintaining resolution and coverage. (**a**) Expansion with a factor of one, where F_1 is formed by F_0; each element in F_1 has a 3 × 3 impact area. (**b**) Expansion with a factor of two, where F_2 is formed by F_1 and each element in F_2 has a 7 × 7 impact area. (**c**) Expansion with a factor of four, where F_3 is formed by F_2; each element in F_3 has a 15 × 15 impact area [33].

Define the receptive field of an element *p* in $F_{i+1}$ as the set of elements in $F_{0}$ that modify the value of $F_{i+1}(p)$. Let the size of the receptive field of *p* in $F_{i+1}$ be the number of these elements. It is easy to see that the size of the receptive field of each element in $F_{i+1}\;$ in $\left(2^{i+2}-1)\times (2^{i+2}-1\right)$. In fact, the area of influence of the square root increases exponentially. More precisely, it can be stated that the number of parameters associated with each layer is the same and the influence area grows exponentially, while the number of parameters grows linearly. This is shown in Figure 4.

#### 3.2.4. Pooling Layer

The next phase is to use the features acquired from the convolutional layer for the purpose of classification. There are no discernible benefits associated with employing a classifier for the purpose of categorizing an extensive array of features. Furthermore, it is possible for the classifier to experience the issue of overfitting. The integration layer is employed as an approach to address this issue, effectively reducing the spatial dimensions and partially mitigating the computational burden associated with network training. It is noteworthy to mention that the merging procedure is executed individually on every image channel. There are two types of integration: maximum integration and mean integration. Peak integration involves determining the highest value inside a certain area by employing an integration window, often a 2 × 2 filter. The practice of average pooling involves computing the mean value within a specified region, and it was initially implemented in the LeNet convolutional neural network. In practical applications, it has been observed that max pooling tends to yield superior results compared to mean pooling due to its ability to retain the discovered characteristics. As a result, the suggested network, which encompasses both the generator and discriminator, employs a strategy of maximum integration.

#### 3.2.5. Group Normalization Layer (GN)

Normalization is a widely employed technique in deep neural networks, which has the potential to enhance the generalizability of the network and facilitate the convergence of the cost function. Normalization is a common practice that is typically carried out subsequent to a fully connected layer, prior to a convolutional layer, or prior to a nonlinear layer. This feature facilitates an accelerated pace of learning and mitigates the model’s dependence on its original configuration. Batch normalization (BN) is a fundamental and valuable technique in the advancement of deep learning, enabling the training of various networks. On the contrary, normalization in relation to the category dimension gives rise to challenges. As the size of the cluster shrinks, there is a significant increase in the BN error. This increase can be attributed to the inaccurate estimation of cluster statistics. As a consequence of this constraint, Bayesian Networks (BNs) are not suitable for training larger models or transferring data to computer vision applications that need the classification of small classes due to their reduced memory utilization. Consequently, the GN approach [21] was chosen as a viable alternative to the BN in this thesis. It is important to acknowledge that this particular layer was only utilized during the productive phase. The Gaussian normalization (GN) technique partitions the channels into several groups and calculates the average and variance for the normalization of each group. The accuracy of GN computations is consistent across a diverse range of batch sizes, indicating that they are not influenced by the size of the batch being processed. The performance of GN is comparable to that of BN when employing standard batch sizes, and it surpasses alternative normalization approaches, such as instance normalization (IN) [34] and layer normalization (LN) [22]. The experimental findings indicate that GN has the potential to effectively substitute BN in various tasks.

Normalization procedures, such as Batch Normalization (BN), Instance Normalization (IN), Layer Normalization (LN), and Group Normalization (GN), typically involve the computation of relational equivalents:

(4) $${\hat{x}}_{i}=\frac{1}{\sigma_{i}}(x_{i}-\mu_{i})$$

where *x* is the feature calculated by a layer and *i* is the index. For example, *i* in a two-dimensional image is a four-dimensional vector as ${i=i_{N}{,i}_{C}{,i}_{H},i}_{W}$ to index the features, where N is the number of samples, C is the number of channels, H is the spatial length, and W is the spatial width. µ and σ represent the mean and standard deviation (std), respectively, and are calculated using the relations:

(5) $$\mu_{i}=\frac{1}{m}\sum_{k\in S_{i}}x_{k}$$

and

(6) $$\sigma_{i}=\sqrt{\frac{1}{m}\sum_{k\in S_{i}}{\left(x_{k}-\mu_{i}\right)}^{2}+\epsilon }$$

**Figure 5:**
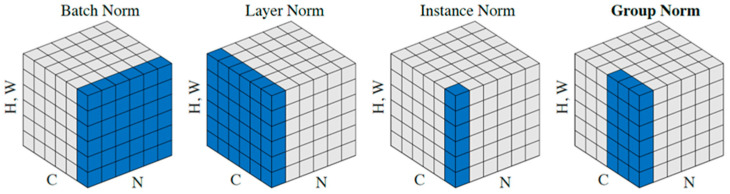
The effectiveness of the BN, LN, IN, and GN normalizing procedures. Each image shows a feature mapping tensor with N representing the number of samples, C representing the number of channels, and (H and W) representing the spatial length and breadth. Blue pixels are normalized with the same mean and variance, which are derived by adding their values [21].

*ϵ* is a small constant to maintain numerical stability, $S_{i}$ is the set of pixels whose mean and standard deviation are calculated, and m is the size of the set. Most of the normalization methods use the relation in common, but they differ in using different pixel sets (in other words, $S_{i}$) to estimate *µ* and *σ*; in other words, the number of their estimated probabilities is different. This is shown in Figure 5. It is worth mentioning that in this form, GN has 2 groups, and each group has 3 channels.

Intuitively, GN uses the set $S_{i}$ to calculate *µ* and *σ*. The set $S_{i}$ has relational equivalents:

(7) $$S_{i}=\left\{k|k_{N}=i_{N},\left\lfloor \frac{k_{C}}{C/G}\right\rfloor =\left\lfloor \frac{i_{C}}{C/G}\right\rfloor \right\}$$

where *G* is a default hyperparameter indicating the number of groups, C/G indicates the number of channels in each group, the ⌊. ⌋ function is correct, and $\left\lfloor \frac{k_{C}}{C/G}\right\rfloor =\left\lfloor \frac{i_{C}}{C/G}\right\rfloor$ indicates that indices *i* and *k* are in the same group of channels. This, of course, is assuming that each group of channels is stored sequentially along the C axis. GN calculates *µ* and *σ* values along H and W and a group of channels (in other words, C/G).

#### 3.2.6. Spectral Normalization (SN)

The instability of the training process is one of the issues in the field of GAN networks. As a result, we applied a unique weight normalization method termed SN [23] to stabilize the discriminator training in this thesis. Among the benefits of this method are its low computational complexity and ease of implementation. On benchmark datasets such as CIFAR-10, STL-10, and ILSVRC2012, the performance of SN has been tested, and it has been experimentally demonstrated that spectrally normalized GANs can produce images of higher or equal quality than previously trained stabilization methods. A simple discriminator based on a neural network with input x can be thought of as the following relationship:

(8) $$f(x,\theta )=W^{L+1}a_{L}(W^{L}(a_{L-1}\left(W^{L-1}(\ldots a_{1}\left(W^{1}x\right)\ldots \right))))$$

where $\theta =\{W^{1},\ldots ,W^{L},W^{L+1}\}$, the set of learning parameters is $W^{l}\in R^{d_{l}\times d_{l-1}}$, and $W^{L+1}\in R^{1\times d_{l}}$ and $a_{1}$ are the non-linear activation function of the drives. 

Here, the bias is omitted for the simplicity of the calculations. The final output of the differentiator can be considered as the relation:

(9) D(x,θ)=Af(x,θ)

where *A* is the activation function associated with the user’s preferred divergence in the distance measurement. The conventional GAN formula is defined as the relationship:

(10) $$\underset{G}{\min}\underset{D}{\max}V(G,D)$$

where the minimum and maximum values of *G* and *D* are determined by assessing the sets of generating and discriminating functions. Goodfellow et al. [9] have proposed the conventional relationship *V (G, D)* in the form of equation:

(11) $$E_{x\sim q_{data}}\left[\log D(x)\right]+E_{x\prime \sim p_{G}}[\log\left(1-D\left(x^{\prime }\right)\right)]$$

where $q_{data}$ is the distribution of the data to be learnt and $p_{G}$ is the distribution of the generator to be trained using min–max adversarial optimization. The assertion of activation function assumes that the value of A in D is a continuous function in [0, 1]. (For example, the sigmoid function.) The optimal discriminant *V (G, D)* for a given generator *G* has the interfaces of the type.

(12) $$D_{G}^{*}\left(x\right):=q_{data}(x)/(q_{data}+p_{G}(x))$$

According to the DL literature, the performance space from which the discriminators are chosen influences the GAN performance. Some studies (such as [24,25,26]) support the necessity of Lipschitz constant continuity in ensuring statistical boundedness. The optimum discriminator of GANs, for example, is produced in the conventional formula as the relation:

(13) $$D_{G}^{*}\left(x\right)=\frac{q_{data}\left(x\right)}{q_{data}+p_{G}\left(x\right)}=sigmoid(f^{*}\left(x\right))$$

$f^{*}\left(x\right)$ is defined as equation:

(14) $$f^{*}\left(x\right)=\log q_{data}(x)-\log p_{G}(x)$$

Also, its derivative is calculated as equation:

(15) $$\nabla_{x}f^{*}\left(x\right)=\frac{1}{q_{data}(x)}\nabla_{x}q_{data}(x)-\frac{1}{p_{G}(x)}\nabla_{x}p_{G}(x)$$

This derivative can be infinite or even uncomputable. This prompts us to apply some regularization conditions to the derivative of *f(x)*.

Refs. [25,26] suggest strategies to regulate the Lipschitz constant of the differentiator by adding a systematic term to input samples *x*. In SN, we followed in their footsteps and sought for the discriminant *D* from continuous K-Lipschitz functions:

(16) $$\underset{{\left\|f\right\|}_{lip}\leq K}{\arg\;\max}V(G,D)$$

where ${\left\|f\right\|}_{lip}$ is the smallest value of M in relation (17) for each x and $x^{\prime }$ with soft L2.

(17) $$\left\|f\left(x\right)-f(x^{\prime })\right\|/\left\|x-x\prime \right\|\leq M$$

Since input-based systematization approaches offer relatively simple formulae based on examples, they suffer from the difficulty that systematization cannot be applied to the space beyond the data generator and distribution without the use of heuristics. The SN technique circumvents this issue by normalizing the weight matrices, as proposed by Yoshida et al. [27]. Following is an explanation of the SN technique in further depth.

The SN method controls the Lipschitz constant of the discriminant function f by limiting the spectral softness of each layer (in other words, $g\;:h_{in}\mapsto h_{out}$). The Lipschitz smooth (in other words, ${\left\|g\right\|}_{Lip}$) is equal to ${sup}_{h}\sigma (\nabla_{g}\left(h\right))$, where σ(A) is the spectral smooth of the matrix A (the L2-soft of the matrix A)

(18) $$\sigma (A)=\underset{h:h\neq 0}{\max}\frac{{\left\|Ah\right\|}_{2}}{{\left\|h\right\|}_{2}}=\underset{{\left\|f\right\|}_{2}\leq 1}{\max}{\left\|Ah\right\|}_{2}$$

This is equivalent to the largest single value of *A*. Therefore, for a linear layer g(h) = Wh, the smooth is obtained using ${\left\|g\right\|}_{Lip}={sup}_{h}\sigma \left(\nabla_{g}\left(h\right)\right)={sup}_{h}\sigma \left(W\right)=\sigma \left(W\right)$. If the Lipschitz smoothness of the activation function ${\left\|a_{l}\right\|}_{lip}$ is equal to 1, we can use the kg inequality to see the following bound on ${\left\|f\right\|}_{lip}$:

(19) $${\left\|f\right\|}_{Lip}\leq {\left\|h_{L}\mapsto {W^{L+1}h}_{L}\right\|}_{Lip}.{\left\|a_{L}\right\|}_{Lip}.{\left\|h_{L-1}\mapsto {W^{L}h}_{L-1}\right\|}_{Lip}\ldots {\left\|a_{1}\right\|}_{Lip}.{\left\|h_{0}\mapsto \;{W^{1}h}_{0}\right\|}_{Lip}=\prod_{l=1}^{L+1}{\left\|h_{l-1}\mapsto {W^{l}h}_{l-1}\right\|}_{Lip}=\prod_{l=1}^{L+1}\sigma \left(W^{l}\right)$$

The SN method normalizes the spectral smoothness of the weight matrix W so that it satisfies the constraint σ(W) = 1 for the Lipschitz constant:

(20) $${\bar{W}}_{SN}(W)=W/\sigma \left(W\right)$$

If we normalize each $W^{l}$ using the relation (20), we can obtain the inequality (), and also we can find that $\sigma ({\bar{W}}_{SN}(W))$ is equal to 1 to find that ${\left\|f\right\|}_{Lip}$ from the upper bound to 1 is limited. The key difference between the SN technique and the soft spectral regularization approach [27] is that the soft spectral approach penalizes the objective function by including an explicit regularization component. This approach differs from the SN method in that it does not attempt to adjust the spectral smoothness to a certain value. In addition, in SN, when the derivative of the normalized cost function is reorganized and the objective function is rewritten, the cost function is augmented with a regularization function depending on the sample data. On the other hand, spectral soft segmentation, such as L2 soft and Lasso segmentation, imposes the independent segmentation of the sample data on the cost function.

#### 3.2.7. Loss Function 

Assuming that the network projections consist of two volumes with the identical resolution as the original input data, it frequently appears that the region of interest occupies a relatively small portion of the volumetric medical images, such as the 3D MRI images employed in this investigation. In this study, the region of interest undergoes processing via a SoftMax layer, which generates the probability of each voxel belonging to either the foreground or background. This occurrence often leads to the learning process getting encased in the local minima of the loss function, which in turn produces a network that exhibits highly biased predictions compared to the background. Consequently, the primary subject matter is typically obstructed or only partially discernible. Previous studies employed loss functions that incorporated sample reweighting, wherein greater emphasis was placed on foreground instances compared to background instances throughout the learning process. Mi-lhtari et al. [11] introduced a novel objective function that utilizes the Dice coefficient, a metric that ranges from 0 to 1, as its basis. The term “*D*” is used to denote the correlation or association between two binary volumes:

(21) $$D=\frac{2\sum_{i}^{N}p_{i}g_{i}}{\sum_{i}^{N}p_{i}^{2}+\sum_{i}^{N}g_{i}^{2}}$$

where collection is performed on *N* voxels, a predicted binary segmentation volume pi∈G, and a binary mask (label) volume gi∈G. This Dice formula can be distinguished by creating a gradient:

(22) $$\frac{\partial D}{\partial p_{j}}=2\left[\frac{g_{j}(\sum_{i}^{N}p_{i}^{2}+\sum_{i}^{N}g_{i}^{2})-2p_{j}\sum_{i}^{N}p_{i}g_{i}}{{\left(\sum_{i}^{N}p_{i}^{2}+\sum_{i}^{N}g_{i}^{2}\right)}^{2}}\right]$$

The prediction is made using voxel *j*. Using this connection, it is not essential to add weights to samples of different classes in order to obtain a reasonable balance of foreground and background voxels, and the experimentally observed results are generated. One of the binary Dice loss function’s key shortcomings is its inability to be used for multi-class segmentation. The mean of the binary Dice function is used as the relation to derive a unique measure for multiclass segmentation:

(23) $$D_{mean}(p,g)=\frac{1}{\left|L\right|}\sum_{1\in L}\frac{2\sum_{i}\left(g_{l}^{i}+p_{l}^{i}\right)}{\sum_{i}(g_{l}^{i}+p_{l}^{i})}$$

where ${\left\{g_{l}^{i}\right\}}_{i\in X,\;l\in L}$ and ${\left\{p_{l}^{i}\right\}}_{i\in X,\;l\in L}$ are the sets of label probability vectors for all real and predicted voxels, respectively. Sudre et al. [29] have proposed a generalized form (*GDL*) of this function. The *GDL* form has a relationship of the form:

(24) $$D_{GDL}(p,g)=\frac{2\sum_{l}\alpha_{l}\sum_{i}min(p_{l}^{i}+g_{l}^{i})}{\sum_{l}\alpha_{l}\sum_{i}min(p_{l}^{i}+g_{l}^{i})}$$

where the coefficient ${\left\{\alpha_{l}\right\}}_{l\in L}$ allows to weigh the participation rate of each class. Since the proposed GAN network includes two independent generators and discriminator structures, we have used two loss functions. The discriminant cost function (LD) consists of two parts. The first part is the sum of the L2 error of the discriminator output, D (.,.), between the original image x and the corresponding mask *y* calculated with a tensor of ones. The second part, the L2 error of the discriminator output between the original image and the prediction of the corresponding segmentation $\hat{y}$, is calculated by the generator with a tensor of zeros; in other words, we have a relation as:

(25) $$L_{D}=L_{2}\left[D\left(x,y\right),1\right]+L_{2}[D\left(x,\hat{y}\right),0]$$

On the other hand, the productive cost function (LG) consists of two parts. The first part is the total L2 error of the discriminator output between the original image *x* and the corresponding segmentation prediction $\hat{y}$ with a tensor of ones. The second part, the GDL error [29], is calculated between the mask image and the output of the generator. In other words, we have interfaces in the form of:

(26) $$L_{G}=L_{2}\left[D\left(x,\hat{y}\right),1\right]+GDL\left(y,\hat{y}\right)$$

## 4. Experiments and Results

### 4.1. Dataset 

**Figure 6:**
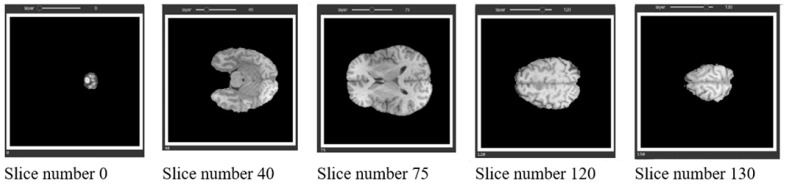
Samples in the dataset.

The approach used in this research involved the utilization of the brain tumor segmentation challenge dataset (BraTs 2021). The dataset was publicly released during the MICCAI conference and was subsequently utilized in a competitive setting, allowing the participating groups to evaluate their novel methodologies using this dataset. The BraTs 2021 dataset comprises three-dimensional magnetic resonance imaging scans of individuals. The Hold-Out strategy was employed to partition the dataset into two distinct subsets, namely the training set and the test set. The training set constituted 80% of the dataset, while the remaining 20% was allocated to the test set. Figure 6 shows the instances inside the dataset, along with a visual representation of their corresponding actual states in a three-dimensional space.

**Figure 7:**
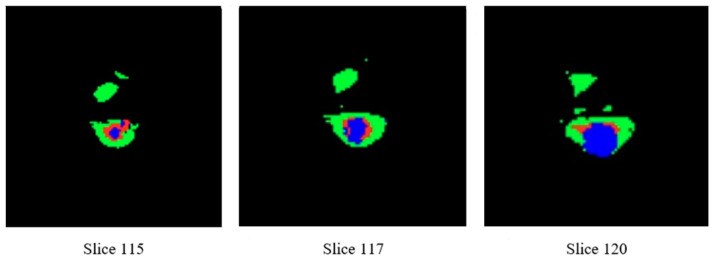
Images of tumor swelling area with three different colors.

The representation of three-dimensional (3D) images on paper is often challenging, thus necessitating the display of the two-dimensional (2D) parts of these images. Figure 7 is an instance from the dataset, whereby three cuts have been made along the three axes of the image. The leftmost column displays the Flair channel images of the sample. The middle column exhibits the equivalent slices extracted from the real-state image. Lastly, the rightmost column portrays the amalgamation of the two columns, revealing three distinct tumor locations on the brain image, each represented by a different color, having undergone a transformation. The provided photos depict the inflamed region of the tumor in a green hue, the necrotic tissue of the tumor in a blue hue, and the actively proliferating core of the tumor in a red hue.

#### Data Set Structure

The BraTS2021 dataset files consist of patients with the highest grade of glioma brain tumor and patients with the lowest grade of glioma brain tumor. There is a separate folder for each patient. The number of patients in the training data set is 1251, and the validation data set includes 219 cases. For each patient, 3D images are available to the researchers; there are four images related to different imaging channels (Flair, T2, T1c, and T1), and one image of a real situation in which different parts of the brain tumor are identified by medical experts. 

### 4.2. Evaluation Criteria

In general, the goal of neural networks is to provide a highly accurate estimate, and in fact, each neural network is an estimator. The closer the value estimated by the network is to the ideal value, the more successful the network is. The type of the estimated value is determined according to the type of ideal labels in the data set. In this research, our ideal labels are three-dimensional images that we call true state images; these images are used in the form of tensors. As a result, the output of our network will be a 3D tensor.

To evaluate an estimator, statistical evaluation criteria are used. The type of statistical criterion is determined according to the nature of the network output and must be chosen in such a way that it can correctly measure the behavior of the estimator to be evaluated.

Our goal in this research is to find the tumor areas in the 3D real-state images. These areas in the real-state tensor are very limited in number because the healthy brain tissues in the real-state image are marked with a zero label and occupy a very large volume of this image. As a result, the estimation of the elements that have a zero label has no value and considering these textures in the evaluation of the results will lead to wrong conclusions. Therefore, the Dice criterion is used in medical image segmentation methods to obtain estimation accuracy because this criterion does not value true negative samples.

Since the proposed GAN network includes two independent generator and discriminator structures, we have used two loss functions. The discriminant cost function (*LD*) consists of two parts. In other words, we have interfaces in the form:

$$L_{D}=L_{2}\left[D\left(x,y\right),1\right]+L_{2}[D\left(x,\hat{y}\right),0]$$

On the other hand, the productive cost function (*LG*) consists of two parts, and we have a relation of:

$$L_{G}=L_{2}\left[D\left(x,\hat{y}\right),1\right]+GDL\left(y,\hat{y}\right)$$

### 4.3. Implementation

Deep neural networks have the capability to undergo training utilizing a central processing unit (CPU). However, due to the substantial computational demands associated with these networks, the training process executed on a CPU tends to be somewhat sluggish and time intensive. The utilization of graphics processing units (GPUs) is prevalent in the training process of the deepest neural networks due to this underlying rationale. The training and testing of the proposed network were conducted on a computer system with 128 GB of RAM, an Intel^®^ Xeon (R) E5-2683 v4 @ 2.10 GHz 52 CPU, and a PNY NVIDIA Quadro P6000 PCI-E Pro 24 GB GPU. Each model took approximately 5–6 days to run. While Google Colab offers the opportunity to utilize the graphics processing unit (GPU) at no cost, it is worth noting that this research also employed its Pro Plus version. However, it is important to acknowledge that the utilization of Google Colab is accompanied by certain obstacles that notably impact the duration of network training. The network was trained using the cross-software framework implemented on the TensorFlow platform. TensorFlow is an open-source software framework that was initially developed by Google and has since been collaboratively enhanced by a global community of contributors. It is primarily implemented using the C programming language. During the implementation phase, the suggested architecture was trained using two different approaches. 

The initial approach involved utilizing images with a higher count of convolutional layers following each normalization. Subsequently, in the subsequent training phase, these layers were omitted in both architectures. Additionally, data augmentation techniques were employed throughout the experiments.

In each training iteration, a systematic process is followed where all the training samples are inputted into the neural network. Subsequently, the network generates an output and evaluates the estimation error. The optimizer function then adjusts the network’s variable parameters. The network’s training duration, when employing data augmentation, averaged approximately 25 min for each training iteration. The training process, which encompassed both methods, required 500 training rounds to achieve the necessary response. Without the inclusion of data augmentation techniques, the full training process lasted around 4–5 days. The network configurations are effectively presented in Table 1.

### 4.4. Results

**Figure 8:**
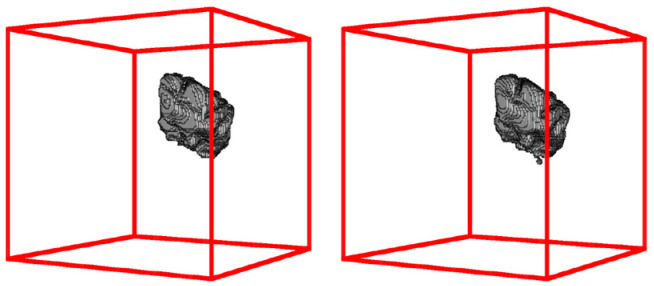
A 3D view of the network output image and the corresponding real-state image.

**Figure 9:**
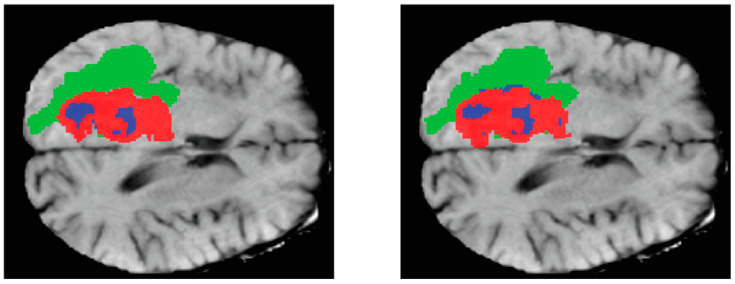
Shows a two-dimensional cross-section of the brain image of one of the existing samples. In this figure, we can see the real status image next to the network output image, and the segmented areas of the brain tumor are marked with three colors. In this way, the swelling area of the tumor is shown in green, the dead tissue of the tumor is shown in blue, and the active core of the tumor is shown in red.

In this study, following the necessary preprocessing procedures specific to magnetic resonance imaging, the obtained data were utilized to generate image segmentation and classification. These outputs were subsequently employed as the input to a proposed network model, aiming to extract either a feature or category. Classifications are employed for categorization purposes. Several studies have demonstrated the potential impact of utilizing deep learning models in conjunction with neural imaging data [33,34]. Additionally, a group of researchers has presented a collection of brain tumor cases [25,29]. The findings presented by the experts indicate a notable enhancement in the classification outcomes. Consequently, it is anticipated that this study will contribute to the advancement of classification results in the domain of brain tumor detection and classification, employing diverse imaging data and deep learning models. Following the training of the proposed network on the majority of the BraTS dataset, we employed various data augmentation techniques to enrich the data and enhance the performance of the network. The subsequent sections will elaborate on the results obtained from these tests, while the final section will include a comparative analysis between the network’s highest accuracy and that of prior research. The initial network configuration is outlined in Table 1. Figure 8 and Figure 9 show a visual depiction of the network’s output and its corresponding steady-state image in three dimensions.

Table 2 and Table 3 summarize the average loss values and accuracy of the two techniques for the overall tumor, the tumor core, and the active tumor core, based on the execution of 1000 batches and 500 IPOCs. Table 3 shows the improved performance due to the second model’s usage of data augmentation and network design.

### 4.5. Comparison of Results

To assess the quality of the suggested approaches, we compared the findings generated from the proposed model with other comparable studies on the BraTS dataset, as shown in Table 4.

Table 3 shows the average accuracy of the two techniques for the three regions of the entire tumor, tumor core, and active tumor core, based on the execution of 1000 batches and 500 IPOCs. As seen in the table, the second model’s usage of data augmentation and network design produced superior outcomes.

## 5. Conclusions

Recent research in the field of medical imaging, specifically focusing on MRI scans, aims to explore various methodologies and their use for the precise extraction of tumor location and classification. This study aimed to implement measures to enhance the following within this field of study. Based on the experimental results of this study, it is recommended that the following methodologies be employed to enhance the precision and caliber of upcoming research attempts. The 3D GAN model that has been proposed exhibits potential for utilization in the upcoming BraTS2021 challenges through multiple avenues. These include training the model to enhance the optimization of the BraTS2021 evaluation criteria, as opposed to relying solely on the criteria provided within the dataset. Additionally, optimizing the proposed architecture can be achieved through the application of algorithmic optimization techniques for hyperparameters. Furthermore, the integration of various data augmentation methods can be explored to enhance the performance of the trained model. The investigation of the multi-task learning approach presents an additional path for inquiry, as it has the potential to be combined with the proposed architecture in order to enhance precision. To enhance the detection and differentiation of tumors, this network has the potential to utilize data from several sources, thereby constructing a more comprehensive representation. The proposed architectural framework can also be assessed using alternative imaging modalities, such as CT scans, and segmentation software designed for general purposes. In conclusion, it can be asserted that the proposed 3D GAN model has the potential to be employed for both image segmentation and image data augmentation. The proposed model has the potential to be employed in conjunction with a 3D noise-to-image GAN model, such as those referenced in citations [38,39,40]. This combination allows for the integration of authentic segmentation outputs, which may then be transformed into realistic MR volumes. The simultaneous utilization of both generative adversarial networks (GANs) shows prospects for the quick production of high-fidelity magnetic resonance (MR) images.

## Figures and Tables

**Table 1:** Network settings.

Learning Rate	1
Optimizer function	Adam
Chance of accidental deletion	30%
Cost	Weighted dice

**Table 2:** Loss values of the proposed methods.

	loss	dice_loss	disc_loss	loss_val	dice_loss_val	disc_loss_val
First Model	2.5476	0.3975	0.5602	2.0057	0.3969	0.0213
Second Model	1.4375	0.2069	0.4030	1.4636	0.2126	0.4004

**Table 3:** Proposed methods accuracy on BraTS2021 dataset.

the Active Tumor Core	Tumor Nucleus	Whole Tumor	Network
0.82	0.82	0.88	Model 1
0.88	0.86	0.94	Model 2

**Table 4:** Comparison of accuracy criteria in different networks on BraTS2021 dataset.

the Active Tumor Core	Tumor Nucleus	Whole Tumor	Network
0.63	0.67	0.85	Ref [35]
0.61	0.72	0.85	Ref [15]
0.73	0.73	0.88	Ref [36]
0.64	0.75	0.89	Ref [37]
0.84	0.86	0.82	Ref [22]
0.84	0.85	0.91	Ref [23]-v1
0.83	0.86	0.92	Ref [23]-v2

## Data Availability

Used dataset is available in: https://www.med.upenn.edu/cbica/brats2021/ and prepared model is available in: https://github.com/hamyadkiani/3D-GAN accessed on 7 September 2023.
